
JOURNAL INFORMATION
==============================
NLM Title Abbreviation: Ann R Coll Surg Engl
Journal ID: Ann R Coll Surg Engl
NLM Journal ID: 7506860
Journal ID: ann

Annals of The Royal College of Surgeons of England

ISSN: 0035-8843
EISSN: 1478-7083
Publisher: Royal College of Surgeons of England

ARTICLE INFORMATION
==============================
PMCID: PMC11163234
DOI: 10.1308/rcsann.2024.0026
Article ID: rcsann.2024.0026
Article version: 1
Subjects: e-Posters

e-Posters

Print publication date: 2024 Mar
Volume: 106
Issue: Suppl 1
First page: S3
Last page: S52
Copyright: Copyright © 2024, The Authors
Copyright year: 2024
License: This is an open-access article distributed under the terms of the Creative Commons Attribution 4.0 International License, which permits unrestricted use, distribution, reproduction, and adaptation in any medium, provided the original work is properly attributed.
License URL: https://creativecommons.org/licenses/by/4.0/

==============================

Subjects: e-Posters, Artificial Intelligence in Surgery

14 Augmented reality reshaping surgical training: trainer and organisational benefits on the path to Kirkpatrick's pyramid mastery

Khalid AW 1 2
Rawaf D 3
ElBahnasawi M 4
Ludick C 5
Harris M 6
Sheikh M 7
Luqman T 8
Beghal G 9
Toms J 9
1 The University of Buckingham , Buckingham, UK
2 Buckinghamshite Healthcare Trust , Stoke Mandeville, UK
3 Inovus Medical , Sutton, UK
4 Manchester University NHS foundation Trust , Manchester, UK
5 Nottingham Trent University , Nottingham, UK
6 Mid Cheshire NHS foundation Trust , Cheshire, UK
7 Surrey and Sussex Healthcare NHS Trust , Surrey, UK
8 Leicester University , Leicester, UK
9 Epsom And St Helier University Hospitals NHS Trust , London, UK

76 AI literature reviews: balancing ends and means

Palagani DSSR
University of Central Lancashire, School of Medicine , Preston, UK

86 A general-purpose natural language processing tool (ChatGPT) can make complex surgical decisions with confidence similar to experienced surgeons: a comparative analysis

Pouris K
Musbahi O
Nurek M
Vella-Baldachino M
Kostopoulou O
Hing C
Bottle A
Cobb J
Jones G
MSK Lab, Imperial College London , London, UK

87 Using artificial intelligence to determine which knee osteoarthritis patients will benefit from steroid injection

Pouris K
Musbahi O
Hadjixenophontos S
Leon D
Soteriou I
Jones G
MSK Lab, Imperial College London , London, UK

185 The application of large language models to orthopaedic practice

Poacher A 1
Caterson J 2
Ambler O 3
Cereceda-Monteoliva N 4
Horner M 5
Jones A 6
1 Cardiff University, Cardiff, UK
2 London School of Hygiene & Tropical Medicine , London, UK
3 Department of Plastic Surgery, Royal Devon and Exeter NHS Trust , Exeter, UK
4 Departement of Plastic Surgery, Guys and St Thomas’ Trust , London, UK
5 Department of Orthopaedic Surgery, University Hospital of Wales , Cardiff, UK
6 Department of Orthopaedic Surgery, University Hospital of Wales , Cardiff, UK

186 Assessing the potential of artificial intelligence for specialist advice: a comparative study with consultant urologists

Smith P
Kailavasan M
Parkinson R
Nottingham University Hospitals NHS Trust , Nottingham, UK

235 The application of deep learning neural networks to surgical simulation and planning: a systematic review

Yasen Z 1
Woffenden H 2
Robinson A 3
Carter G 4
Bello F 5
1 Imperial College University , London, UK
2 Ministry of Defence , Birmingham, UK
3 Lewisham and Greenwich NHS Trust , London, UK
4 Barts Health NHS Trust , London, UK
5 Imperial College London , London, UK

278 A review of novel techniques and most recent uses of artificial intelligence/machine learning in orthopaedic surgery

Patel J 1
Jones D 2
1 University of Oxford , Oxford, UK
2 Gleamer, Paris, France

Subjects: Clinical Research

19 Efficacy of REZUM on chronic retention: initial experience in a district general hospital

Pendegast H 1
Bibby L 1
AbuSanad O 1 2
1 Chesterfield Royal Hospital , Chesterfield, UK
2 Sheffield Teaching Hospitals , Chesterfield, UK

33 Ureteric calculus in a left complete duplex system masquerading as an impacted stone

Adenipekun A
Gaballa N
Darrad M
University Hospitals Birmingham NHS Foundation Trust , Birmingham, UK

41 Postoperative surgical site infection following laparotomy: PICO dressings vs standard wound dressings

Murphy Lonergan R 1 2
Singh A 3
Habib Bedwani N 2 4
1 Chelsea and Westminster Hospital NHS Foundation Trust , London, UK
2 Imperial College London , London, UK
3 St George's University , St. George's, UK
4 Barts Health NHS Trust, London, UK

59 The safety of glycopeptide impregnated calcium sulphate following debridement implant retention and antibiotics (DAIR) of the infected total knee replacement

Hamal P 1
Raven-Gregg T 2
Barksfield R 3
Porteous A 4
Murray J 4
1 University of Bristol , Bristol, UK
2 Cardiff University , Cardiff, UK
3 Gloucestershire Hospitals NHS Foundation Trust , Gloucester, UK
4 North Bristol NHS Trust, Bristol, UK

71 Evaluating ENT junior doctors’ proficiency in interpreting audiograms and tympanograms: a cross-sectional survey

Patel S 1
Siddiqui Z 2
Dusu K 3
Shah S 4
1 Leicester Royal Infirmary, Leicester, UK
2 Frimley Park Hospital , Frimley, UK
3 Surrey and Sussex Healthcare NHS Trust, Redhill, UK
4 Tunbridge Wells Hospital , Tunbridge Wells, UK

112 Cystolithotripsy under local anaesthetic – an effective treatment option for comorbid patients with encrusted ureteric stents

McComb K 1
Tomalieh F 2
El-Ali A 2
Calleary J 2
1 North Manchester General Hospital , Manchester, UK
2 Fairfield General Hospital, Manchester, UK

129 Elective amputations as a sequelae of lower limb trauma: rationale for amputation and long-term complication rates

Linden A
Brookes C
Dardak S
Qayyum-Bin-Asim M
Trompeter A
St. George's Hospital, London, UK

140 An analysis of colorectal cancer surgery waiting times during and after COVID-19 lockdown in a regional tertiary centre

Enc ME
Gowda MS
Venkatesan G
Thulasiraman S
Cherian Peter B
South Tees NHS Foundation Trust, Middlesbrough, UK

141 Factors influencing Novosorb integration and complications in non-burn wound reconstruction: retrospective observational study

Kolaityte V
Kidd T
O'Hara N
Bechar J
Izadi D
Wallace D
Bajaj K
UHCW , Coventry, UK

142 The change in practice of aortic procedures following the introduction of hybrid theatre in a tertiary centre

Thiagarajan J 1
Ninkovic-Hall G 1
Neequaye S 2
1 University of Liverpool , Liverpool, UK
2 Royal Liverpool Hospital , Liverpool, UK

149 Review of natural history and management of gallstone ileus: case series

Kumar S
Basildon Hospital, Basildon, UK

163 Predicting difficult laryngeal exposure (DLE) during operative microlaryngoscopy: evaluating the predictive value of Laryngoscore in a single centre UK population in a Scottish teaching hospital

Haider A 1
Shakeel M 2
1 Aberdeen University , Aberdeen, UK
2 Aberdeen Royal Infirmary, Aberdeen, UK

165 Laparoscopic management of adhesional small bowel obstruction: Is it feasible?

Hadjicosta O
Spyropoulou L
Hamza Y
Ubaide HN
Aker M
Arulampalam T
Colchester General Hospital, Essex, UK

167 Follow-up after successful Pavlik harness treatment for DDH: Is two years enough?

Larwood J
Idowu O
Lindisfarne E
Elliott K
Aarvold A
University Hospital Southampton , Southampton, UK

168 Shenton’s line in DDH: Useful or useless?

Larwood J
Hasan W
Connell R
Lindisfarne E
Elliott K
Aarvold A
University Hospital Southampton , Southampton, UK

171 Meta-analysis of randomised controlled trials comparing laparoscopic with open mesh repair of recurrent inguinal hernia

Manasseh M 1 2
1 Ain Shams University , Cairo, Egypt
2 Torbay and South Devon NHS foundation Trust, Torquay, UK

177 Improving the detection of low-grade appendiceal mucinous neoplasms (LAMNs) to reduce pseudomyxoma peritonei (PMP) risk

Golash A
University of Buckingham , Buckingham, UK

179 Effectiveness of mini-laparotomy vs laparoscopic cholecystectomy: an updated meta-analysis of 2,964 cholelithiasis patients

Mithany R 1
Abdallah S 2
1 Kingston Hospital NHS Foundation Trust, Kingston Upon Thames , UK
2 Jaber Al-Ahmad Hospital, Kuwait, Kuwait

211 COALA Trial: changing outcomes after low anterior resection in colorectal cancer by anal sphincter prehabilitation prior to reversal of ileostomy – a parallel explanatory (COALA-1) and pragmatic trial (COALA-2) protocol

Ong HI 1 2
Chen WY 2 1
Tisseverasinghe S 1
Nadarajah R 3 4
Pollard C 5
Barr R 5
Carrington E 5
Malcolm A 6 7
Fleming C 8
Proud D 1 2
Burgess A 1 2
Mohan H 1 9 2
1 Austin Health, Melbourne, Australia
2 University of Melbourne , Melbourne, Australia
3 Royal North Shore Hospital , Sydney, Australia
4 Westmead Hospital , Sydney, Australia
5 Imperial College Healthcare , London, UK
6 North Shore Private Hospital , Sydney, Australia
7 University of Sydney , Sydney, Australia
8 University Hospital of Limerick , Limerick, Ireland
9 Peter MacCullum Cancer Center, Melbourne, Australia

231 Evaluation of use, cost, and carbon footprint impact of routine histopathological analysis of anastomotic doughnuts following colorectal surgery: a 3-year review

Moussa R
Ur Rehman M
Siaw Lin C
Ahmed J
Northampton General Hospital , Northampton, UK

239 Outcomes of dual mobility bearings in revision total hip replacements: a consecutive single-surgeon case series

White C
Abdalla W
Subramanian P
Royal Free London NHS Trust , London, UK

245 Fracture-related infection in the NHS: an analysis of healthcare utilisation and costs

Woffenden H 1
Yasen Z 2
Burden E 1
Douthwaite A 1
Elcock S 1
Mclean L 1
von Hoven P 1
Fenton P 1
1 Queen Elizabeth Hospital , Birmingham, UK
2 Barts Health NHS Trust , London, UK

246 Segmentectomy or wedge resection in pulmonary metastatectomy

Badran A 1
Jaffar-Karballai M 2
Sheikh-Yasin K 1
Amer K 1
Alzetani A 1
1 Southampton University Hospital , Southampton, UK
2 St George's University of London , London, UK

248 Replantation of amputated right ring finger secondary to avulsion injury: a unique case exploring microsurgical principles in hand trauma

Morrison C 1 2
Al-Hashemi S 2
1 St Vincent's University Hospital , Dublin, Ireland
2 University College Dublin , Dublin, Ireland

250 Topical tranexamic acid wash to reduce blood in total hip arthroplasty - do the NICE guidelines work?

Patel J 1
Abhee S 2
Slater G 2
Ahmed S 2
1 University of Oxford , Oxford, UK
2 Maidstone and Tunbridge Wells NHS Trust , Kent, UK

253 Predicting rotator cuff tears in patients with shoulder dislocations combined with an isolated fracture of the greater tuberosity by means of radiographic characteristics

White C
El-Gendy M
Elshafey A
Makki D
West Hertfordshire Teaching Hospitals NHS Trust , Watford, UK

274 Assessing the feasibility and outcomes in laparoscopic nephrectomy of large renal masses in complex patients treated with renal artery pre-embolisation

Sooltangos A
Abdul R
Elfar M
Venugopal S
Royal Liverpool University Hospital , Liverpool, UK

277 How safe is it to follow-up Bosniak 2 cysts?

Sooltangos A
Elfar M
Royal Liverpool University Hospital , Liverpool, UK

Subjects: Collaborative Research

126 Assessing the impact of a collaborative online teaching series by students and surgeons on enhancing clinical anatomy proficiency among medical students

Fang S
Subbiah Ponniah H
Edmiston T
Lloyd-Davies C
Wellington J
Royal Society of Medicine, London, UK

210 Developing a prehabilitative pelvic floor muscle training programme for prevention of low anterior resection syndrome in rectal cancer patients

Ong HI 1 2
Tisseverasinghe S 1
Nadarajah R 3 4
Pollard C 5
Barr R 5
Carrington E 6
Malcolm A 7 8
Fleming C 9
Proud D 1 2
Burgess A 1 2
Mohan H 1 10 2
1 Austin Health, Melbourne, Australia
2 University of Melbourne , Melbourne, Australia
3 Westmead Hospital , Sydney, Australia
4 Royal North Shore Hospital , Sydney, Australia
5 Imperial College Healthcare , London, UK
6 Imperial College Healthcare , London, UK
7 North Shore Private Hospital , Sydney, Australia
8 University of Sydney , Sydney, Australia
9 University Hospital Limerick , Limerick, Ireland
10 Peter MacCullum Cancer Center, Melbourne, Australia

Subjects: Early-Stage Innovation

4 The role of three-dimensional printing in person specific implants following traumatic injury

Howard J 1 2
Ahmed Z 2
1 University of Liverpool Medical School , Liverpool, UK
2 University of Birmingham, Medical School , Birmingham, UK

58 Ethics and AI in surgery: unveiling ChatGPT's promise and perils – a comprehensive review

Locurcio LL
NHS , Newcastle, UK

100 ERCP and plastic biliary stenting for choledocholithiasis: an assessment of outcomes

Gower D
Curwen O
Nehra D
Hwang R
St. Helier Hospital, London, UK

106 Grade II and grade III oligodendroglioma and astrocytoma – peripheral blood profiling

Tsai PH
Roncaroli F
Manchester University , Manchester, UK

132 Use of sensors and their role in orthopaedics

Kamal J
Cambridge University Hospitals NHS Foundation Trust , Cambridge, UK

134 Enhancing non-burn wound management with Novosorb: a comprehensive case series

Kolaityte V 1 2
Kidd T 1
O'Hara N 1
Bechar J 1
Izadi D 1
Bajaj K 1
Wallace D 1
1 UHCW , Coventry, UK
2 GSTT , London, UK

136 Objective measurement of fluid extravasation following hip arthroscopy

Kamal J
Cambridge University Hospitals NHS Foundation Trust , Cambridge, UK

178 Building a digital clinical community in UK hospitals for collaboration, networking and peer support

Starostina A
University of Nottingham , Nottingham, UK

192 Critical analysis of GPT-4’s ability to pass the MRCS Part A examination

Haq I 1
Raj S 1
Ridha A 2
Syed F 1
O’Sullivan A 1
Ahmed I 1
Syed F 1
Khatri C 1
1 University Hospitals Coventry & Warwickshire , Coventry, UK
2 Warwick Clinical Trials Unit, Clinical Sciences and Research Laboratories , Warwick, UK

240 Uncommon peritoneo-thoraco-cutaneous fistula post complex cholecystectomy surgery: a case report

Khalid MA 1 2
Mallon C 1
Abdalkarem Abdalla A. Mahgoub 1
El Madani S 1
Ali H 1
Marzouk A 1
1 Altnagelvin Area Hospital , Londonderry, UK
2 Ulster University School of Medicine , Londonderry, UK

244 Same day hip arthroplasty: a reality

Patel J 1
Ahmed S 2
1 University of Oxford , Oxford, UK
2 Maidstone and Tonbridge Wells NHS Trust , Kent, UK

259 Advanced preoperative planning techniques in the management of complex proximal humerus fractures

Yasen Z
Imperial College London , London, UK

266 Rethinking knee osteoarthritis treatment: efficacy and cost of PRP vs corticosteroids in the NHS context

Yasen Z
Imperial College London , London, UK

Subjects: Global surgery (at least one author from a low- and middle-income country (LMIC))

21 A scoping review on management of male infertility in Africa

Abu M
Davis SI
Abdullahi Z
Igbokwe M
Adamu-Biu F
Oluwadamilola A
Saleh N
Ikuborije P
Olalekan A
Abimbola O
Mohammed T
Ojo C
Udoji C
Nwadinigwe E
Adenipekun A
Adewale O
Uduh C
Oladapo O
Haladu Z
Olumide N
Oyindamola A
Surgery Interest Group of Africa , Lagos, Nigeria

27 A rare presentation of intestinal intussusception in a child with Peutz-Jeghers syndrome

Ovechkin D 1
Awuah WA 1
Wellington J 2
Moskalenko R 1
Dmytruk S 1
Abdul-Rahman T 1
Ovechkina Y 1
Bharadwaj H 3
1 Sumy State University , Sumy, Ukraine
2 Bradford Teaching Hospitals NHS Foundation Trust , Bradford, UK
3 The University of Manchester , Manchester, UK

48 Neuroendoscopy and global neurosurgery: a narrative review of the diversity of minimally invasive procedures in pituitary surgery

Lee KBC 1
Wellington J 2 1
1 Cardiff University School of Medicine , Cardiff, UK
2 Bradford Teaching Hospitals NHS Foundation Trust , Bradford, UK

146 Advancing awareness: examining the impact of gender equity conferences in global surgery communities

Rabiee P 1 2 3
Faria I 4 3
Shukla S 5 3
Jose AM 6 3
Gerk A 7 3
Campos LN 8 3
1 Hull Teaching Hospital , Hull, UK
2 Royal London Hospital , London, UK
3 Gender Equity Initiative in Global Surgery , Boston, USA
4 University of Texas Medical Branch , Galveston, USA
5 Qingdao University College of Medical Science , Qingdao, China
6 Datta Meghe Institute of Medical Sciences, Sawangi, India
7 McGill University , Montreal, Canada
8 Universidade de Pernambuco , Recife, Brazil

164 Gastroschisis in sub-Saharan Africa: a scoping review of the prevalence, management practices, and associated outcomes

Zubair A
Fatona O
Opashola K
Faleye A
Adeyanju T
Adekanmbi A
Etiubon E
Jesuyajolu D
Surgical Interest Group , Africa, Lagos, Nigeria

180 Biliary reconstruction in paediatric living donor liver transplantation: a systematic review and meta-analysis

Elkomos BE 1 2
Alkomos PE 3
Alkomos M Fr 4
Ebeidallah G 5
Abdelaal A 6
1 General and Emergency Surgery Department, Northwick Park Hospital, London Northwest Hospital NHS Trust , London, UK
2 General Surgery Department, Faculty of Medicine, Ain Shams University , Cairo, Egypt
3 Faculty of Medicine, Ain Shams University Hospital , Cairo, Egypt
4 Gastroenterology Department, St. Joseph’s University, Paterson , New Jersey, USA
5 Emergency Department, Royal Derby Hospital, University Hospitals of Derby and Burton NHS Foundation Trust , Derby, UK
6 General Surgery Department, Faculty of Medicine, Ain Shams University , Cairo, UK

181 Hepatic artery anastomosis in living donor liver transplantation: systematic review and meta-analysis

Elkomos BE 1 2
Alkomos PE 3
Alkomos MF 4
Elsaigh M 1
Sohail A 1
Awan B 1
Ebeidallah G 5
Abdelaal A 6
1 General and Emergency Surgery Department, Northwick Park Hospital, London Northwest Hospital NHS Trust , London, UK
2 General surgery department, Faculty of Medicine, Ain Shams University , Cairo, Egypt
3 Faculty of Medicine, Ain Shams University Hospital , Cairo, Egypt
4 Gastroenterology Department, St. Joseph’s University , Paterson, New Jersey, USA
5 Emergency Department, Royal Derby Hospital, University Hospitals of Derby and Burton NHS Foundation Trust , Derby, UK
6 General Surgery Department, Faculty of Medicine, Ain Shams University , Cairo, Egypt

200 Computerised tomography scan in acute appendicitis: a diagnostic necessity or first world privilege?

Chang LMY 1
Nadarajan D 2
1 Hull University Teaching Hospitals NHS Trust , Hull, UK
2 Hospital Seri Manjung , Manjung Perak, Malaysia

212 Working together to achieve more than surgical procedures. a longstanding collaboration with a single centre in rural Ghana

Watt J 1
Acquah E 2
1 Operation International, London, UK
2 Holy Family Hospital , Techiman, Ghana

224 Incidence of burst abdomen and associated patient factors in Bangladesh – a multicenter study

Noor M 1
Tamanna R 2
Jahan N 3
Ahmad M 4
Ahmad WU 4
1 Queen Alexandra Hospital , Portsmouth, UK
2 Watford General Hospital , Watford, UK
3 Ad-din Barrister Rafiqul Huq Hospital , Dhaka, Bangladesh
4 Sir Salimullah Medical College and Mitford Hospital , Dhaka, Bangladesh

255 A systematic review and meta-analysis on outcomes of valvular heart surgery in Africa

Akintoye O
Abdulmalik M
Usamah B
Olakanmi D
Gyau-Ampong C
Surgery Interest Group of Africa, Lagos , Nigeria

258 E-cigarettes (vaping) and peripheral vascular disease: a literature review and recommendations

EL-Nakhal T 1
Alhindawi B 2
Al-Najjar A 2
Taani B Al 3
Sadat U 4 5
1 Nottingham University Hospitals , Nottingham, UK
2 Al-Azhar University Gaza , Gaza, Palestine
3 Leicester University Hospitals , Leicester, UK
4 Cambridge Mathematics of information in Health Hub, Cambridge, UK
5 Lister Hospital , East and North Hertfordshire NHS Trust, Stevenage, UK

264 Predictors of catastrophic cost of motorcycle-related road traffic injuries: experience from a major trauma reference centre in a lower-middle income country

Oladeji E 1 2
Ezeme C 1 2
Baiyewu L 1
Okunlola M 1
Ogunlade S 1
1 Department of Surgery, University College Hospital , Ibadan, Nigeria
2 St Richard's Hospital , Chichester, UK

265 Outcomes of coronary artery bypass graft surgery in Africa: a systematic review and meta-analysis

Akintoye O
Fasina O
Adiat T
Nwosu P
Olubodun M
Adu B
Surgery Interest Group of Africa, Lagos, Nigeria

275 Disparities in the clinical profile of spinal tuberculosis in Africa: a scoping review of management and outcome

Oladeji E
Enemuo T
Anthony-Awi T
Olaniyi A
Olaku J
Aransiola P
Salawu R
Adedoyin G
Olatide O
Surgery Interest Group of Africa, Lagos, Nigeria

280 Perforated peptic ulcer disease: a scoping review of the Nigerian experience

Obuh O 1
Egede F 2
Sydney R 1
1 Surgery Interest Group of Africa, Lagos, Nigeria
2 University Of Benin , Benin , Nigeria

Subjects: QI and audit

2 Can we improve the emotional support provided to FY1 doctors?

McGowan K
SHSCT , Belfast, UK

6 Transmetatarsal amputation: a chart review of its outcome over the past eight years

Nwatuzor C
Syed M
Siddiqui T
University Hospital Hairmyres , Hairmyres, UK

7 Developing a structured digital proforma to improve documentation standards in general surgery ward rounds

Wedlich M 1 2
Adi R 2
1 Addenbrookes Hospital , Cambridge University Hospitals NHS Foundation Trust , Cambridge, UK
2 Hinchingbrooke Hospital , North West Anglia NHS Foundation Trust, Huntingdon, UK

9 A closed-loop audit of diabetic medication and GKI prescriptions for surgical patients in a general surgery service

Lai L
The James Cook University Hospital , Middlesbrough, UK

10 A closed loop quality improvement project: the pain ladder and the scalpel

Naathan H 1
Gundle L 2
Pickering D 3
1 Queens Medical Centre , Nottingham, UK
2 GSTT, London, UK
3 Quotient Sciences, Nottingham, UK

20 An audit of bone protection prescribing in patients with fragility fractures in the primary care setting

Redman I 1
Sivanesan V 2
1 Ealing Hospital , London North West University Healthcare , NHS Foundation Trust, London, UK
2 Mansell Road Practice, London, UK

23 The neck of femur fractures admission pathway: improving patient safety and flow

Gupta S
Aslam Z
Bokari A
Royal Blackburn Hospital , Blackburn, UK

24 Sweet spot or blind spot? A review of diabetes detection in anorectal abscess patients

Karedath J
Smith C
Dhana R
Dhungana A
Helmy A
Chalabi H Al
Princess Royal University Hospital , King's College Hospital NHS Foundation Trust , London, UK

26 A retrospective investigation on the relationship between PCNL waiting times and the likelihood of kidney stone complications and the impact of the COVID-19 outbreak on PCNL waiting lists

Rosli MD Bin
United Lincolnshire Hospital NHS Trust , Lincoln, UK

31 Simulation-based teaching to bridge the gap between IMGs and UK graduates - a QIP

Musai J 1
Yeldham F 2
1 Imperial College Healthcare NHS Trust , London, UK
2 Chelsea and Westminster NHS Foundation Trust, London, UK

34 Improving length of stay following transurethral resection of bladder tumour in a local urology unit

Adenipekun A 1
Jallad S 2
1 University Hospitals Birmingham NHS Foundation Trust , Birmingham, UK
2 Frimley Health NHS Foundation Trust, Slough, UK

36 H. Pylori eradication therapy for the patient who underwent emergency surgery for perforated peptic ulcer disease

Maula K
Eddama M
Dalip A
Nixon J
East Kent Hospitals University Foundation Trust , Margate, UK

37 Introduction of ambulatory USS slots for patients attending the surgical admissions unit

Stylianou T
Yershov D
Hirri F
Findlay L
Salisbury District Hospital , Salisbury, UK

40 NELA postoperative geriatrics review audit

Song M 1 2
Oyewole B 1
Cheek C 1
1 Hereford County Hospital , Hereford, UK
2 Worcestershire Royal Hospital , Worcester, UK

42 "Hip Hip Hooray”: Ensuring sufficient analgesia and bone protection for patients with hip fractures

Al Hajaj S
Rayan F
Markose B
Kettering General Hospital , Kettering, UK

43 The impact of the COVID-19 pandemic on the management of hip fractures against best practice tariff key criteria a retrospective study

Al Hajaj S
Ward T
Vlazaki M
Rayan F
Shyamsundar S
Kettering General Hospital , Kettering, UK

47 Introduction of an ENT admission proforma to improve documentation and provide quality patient care

Odedra A
Ahmed T
Cheung K
Sanders O
Ioannidis D
Colchester General Hospital , Colchester, UK

51 Reducing on the day elective plastic surgery theatre list cancellations

Ramjeeawon A
Neves P
Bulstrode N
Great Ormond Street Hospital , London, UK

53 The journey to surgical sustainability: assessing the impact of green theatre practice through carbon footprint analysis

Walshaw J 1
Westwood E 2
Boag K 1 2
Chua WY 3
Dimashki S 2
Khalid H 2
Lathan R 4
Wellington J 2
Yiasemidou M 5
1 Leeds Institute of Medical Research , St James’s University Hospital , University of Leeds , Leeds, UK
2 Bradford Teaching Hospitals NHS Foundation Trust , Bradford, UK
3 University of Nottingham , Nottingham, UK
4 Hull University Teaching Hospitals NHS Trust , Hull, UK
5 Oxford University Hospitals NHS Foundation Trust , Oxford, UK

54 Quality and readability of online information and materials on seromas

Bald A
Gateshead NHS Foundation Trust , Newcastle, UK

55 The age of quilting: a retrospective study investigating seroma formation following a mastectomy with quilting

Bald A
Fasih T
Pounder L
Gateshead NHS Foundation Trust , Newcastle, UK

62 Adhesional small bowel obstruction (ASBO): Is it time to change our practice?

Karagiannidis G
Khaliq T
El-Farran M
Labinoti R
Youssef F
Ipswich Hospital NHS Trust , Ipswich, UK

63 Testicular torsion in a district general hospital, a 5-year review

Hoo ZL 1
Wong YW 2
Simpson L 3
1 Hull University Hospitals NHS Trust , Hull, UK
2 Manchester University Hospital NHS Foundation Trust , Manchester, UK
3 Leeds Teaching Hospitals NHS Trust , Leeds, UK

64 PIRADS mpMRI: positive predictive value of pre-biopsy prostate MRI

Wong YW 1
Hoo ZL 2
1 Manchester University Hospital NHS Foundation Trust , Manchester, UK
2 Hull University Teaching Hospitals NHS Trust , Hull, UK

66 Assessing serum calcium and uric acid level in patients with ureteric stones

Adenipekun A 1
James N 2
Aldiwani M 2
1 University Hospitals Birmingham NHS Foundation Trust , Birmingham, UK
2 Frimley Health NHS Foundation trust, Slough, UK

68 No teaching today – no surgeons tomorrow: structured tutorials improve medical students’ confidence and competence in specialist surgical fields

Dhas K
Murphy Lonergan R
Bartholomew B
Chelsea and Westminster Hospital NHS Foundation Trust , London, UK

69 Outcomes following hemithyroidectomy and exploring factors that may contribute to length of stay

Nuredini G
Soltan I
Zhang H
Barts Health NHS Trust , London, UK

72 How efficient are we when it comes to the use of incentive spirometry across the surgical floor?

Alaeq A
Zino S
Ninewells Hospital , Dundee, UK

77 Audit of the quality of consent form completion for cholecystectomies performed at a tertiary centre in the UK

Patel S
Arshad A
University Hospital Southampton NHS Foundation Trust , Southampton, UK

80 An audit on improving compliance of oxygen prescribing in the ENT department

Rasquinha M
Bonduelle Q
Sethi N
Nottingham University Hospital NHS Trust , Nottingham, UK

82 An audit on improving documentation of management in patients presenting with otitis externa to the ENT emergency clinic

Rasquinha M
Bonduelle Q
Abbas Y
Cho W
Nottingham University Hospital NHS Trust , Nottingham, UK

84 Evaluation and improvement of TURBT operation notes in a district general hospital

Challapalli P
Maniarasu S
Samian D
Mistry R
Elsayed M
Southport District General Hospital , Liverpool, UK

89 Factors affecting ileostomy reversal time at ELHT – 5-year observational study

Sayed MA 1
Heywood NA 1
Nicholson J 2
Hardcastle G 2
1 Royal Blackburn Teaching Hospital , Blackburn, UK
2 Lancaster Medical School , Lancaster, UK

92 Intraoperative use of tourniquet in trauma and orthopaedics

Ponsworno KV
UHBW NHS Trust, Bristol, UK

93 A multi-cycle closed loop audit looking at the documentation and positioning of intercostal chest drains (ICD’s) in trauma patients presenting to a major trauma centre

Durrant J
Royal London Hospital , London, UK

98 Optimising perioperative management of microvascular breast free flaps: a closed-loop audit

Frommer ML 1 2 3
Walker C 3
Karoshi A 4
Jasionowska S 1 3
Langridge BJ 1 2 3
Nikkhah D 3
Hamilton S 3
1 The Charles Wolfson Centre for Reconstructive Surgery , London, UK
2 UCL Department of Surgical Biotechnology, Division of Surgery and Interventional Science, London, UK
3 The Royal Free London NHS Foundation Trust, London, UK
4 UCL Medical School , London, UK

99 Consent in hand trauma surgery

Ramjeeawon A
Mendis K
Toft N
Royal Free Hospital , London, UK

102 Improving staff knowledge of the penetrating neck trauma guideline at a major trauma centre: a quality improvement project

Stavropoulou-Tatla S 1
Awal D 1
Singh N 1
Fardanesh A 2
Ryba F 1
1 King's College Hospital , London, UK
2 Whipps Cross Hospital , London, UK

103 Vacuum-assisted mini-percutaneous nephrolithotomy (Mini-PCNL) with ClearPetra sheath: initial experience and outcomes

Ho B
Pai A
Northampton General Hospital , Northampton, UK

104 Ureteric stent passport: improving patient stent knowledge and care

Ho B
Trerattanavong K
Kasbia H
Cheung C
Pai A
Northampton General Hospital , Northampton, UK

105 Ventriculoperitoneal shunt dysfunction in children with constipation

Mohammed N 1
Sunny JT 2
Garnett M 3
Naushahi M 3
1 Gonville & Caius College , University of Cambridge , Cambridge, UK
2 National Hospital for Neurology and Neurosurgery , London, UK
3 Addenbrooke's Hospital , Cambridge, UK

107 Clinical audit to improve the current practice management of discitis

Shahul Hameed MR
Singh NKP
Ashford and ST. Peter's NHS Foundation Trust , Surrey, UK

111 Does Liquiband skin adhesive offer a safe and effective alternative to traditional suturing in the closure of paediatric circumcision wounds?

McComb K 1
Ravindranath N 2
Surange R 2
1 North Manchester General Hospital , Manchester, UK
2 Royal Oldham Hospital , Manchester, UK

114 Adherence of trauma and orthopaedics operative notes to the RCS Good Surgical Practice Guidelines

Shahul Hameed MR
Yogarajah T
Syed T
Mathew F
Shaunak S
Ashford and St. Peter's NHS Foundation Trust , Surrey, UK

117 'Home in Time' early surgical discharge quality improvement project

Angamuthu N
Boyle D
Hart C
Royal Free Hospital , London, UK

118 Audit of compliance of VTE prophylaxis guidelines (NICE and ACPGBI) after colorectal cancer resections

Angamuthu N
Varcada M
Ogunbiyi O
Royal Free Hospital , London, UK

119 Acute uncomplicated diverticulitis – do we treat it right?

Karagiannidis G
Khadra T
Battol SN
Muralidharan E
El-Farran M
Pitt J
Ipswich Hospital NHS Foundation Trust , Ipswich, UK

122 Routine pre-scheduled ‘Case of the Week’ surgical teaching improves confidence of foundation trainees in management of surgical patients: a completed audit cycle

Bhojwani A
Gorgoraptis S
Golash A
University Hospitals of North Midlands NHS Trust , Stoke-on-Trent, UK

123 Audit on compliance of LAOPS checklist in minor operations in the plastics surgery department of lister: to prevent zero events

Acharya P
East and North Hertfordshire NHS Trust , Stevenage, UK

124 Spontaneous stone passage of ureteral stones in patients with a percutaneous nephrostomy for acute ureteric obstruction

Bhojwani A
Gorgoraptis S
Sarmah P
Fernando H
University Hospitals of North Midlands NHS Trust , Stoke-on-Trent, UK

125 Residual volume and DRE/PV documentation in patients presenting with urinary retention – a closed loop audit

Tomalieh F
Royal Oldham Hospital , Oldham, UK

127 ReSPECT form compliance in a major trauma centre’s orthogeriatric cohort - a closed loop audit

Ghandour O
Smith I
Kelly E
Berry A
Everett S
North Bristol Trust, Bristol, UK

128 A review of emergency general surgery (EGS) admissions via the direct access pathway (DAP) in a district general hospital

Pal S 1
Bartolome IA 2
Pangeni A 1
Kamal Nahid MA 1
Shrestha A 1
1 East Kent Hospitals University NHS Foundation Trust , Ashford, UK
2 University Hospital Sussex NHS Foundation Trust , Brighton, UK

131 Colonoscopy audit – are we collecting the correct biopsies?

Kamal J
West Suffolk NHS Hospital , Bury St Edmunds, UK

133 Audit to improve Lister Hospital's performance on National Hip Fracture Database (NHFD) in regard to the operative management of extracapsular neck of femur fractures

Acharya P
Lister Hospital , Stevenage, UK

135 A closed-loop audit to improve compliance with the liver shrinkage diet in elective cholecystectomy patients with high BMI

George J
The Shrewsbury and Telford Hospital NHS Trust , Shrewsbury, UK

138 Assessing effect of surgical skills teaching on clinician morale

Shaharudin A
King's College Hospital , London, UK Royal London Hospital , London, UK

139 Investigating the accuracy of electronic patient record systems in general surgery operations – a tertiary centre audit first phase quality improvement project

Nightingale K 1
Arain J 2
1 MBChB, Leeds, UK
2 MB BS, Bahawalpur, Pakistan

151 Paperless handover: an effective measure worth taking

Abdulshafea M 1
Topliss C 2
1 University of Central Lancashire , Preston, UK
2 Swansea Bay Health Board, Swansea, UK

152 Surgical site infection after hip fracture surgery: a prospective follow up audit of monocryl wound closure

Abdulshafea M 1
Amjad A 2
Altahir A 2
Porter K 2
Mohammed A 2
1 University of Central Lancashire , Preston, UK
2 Swansea Bay Health Board, Swansea, UK

155 Using artificial intelligence models to reduce prescribing errors in surgery: a large multi-centre audit

Fricker M 1
Khosa S 2
Islam S 2
Jawad Z 2
1 Imperial College London , London, UK
2 London North West University Healthcare NHS Trust , London, UK

156 Sepsis form in patients present to Whipps Cross Hospital

Mousa H
Barts Health NHS Trust, London, UK

158 Enhancing adherence to BSSH guidelines for hand injuries requiring surgery: a two-cycle audit

Yip S
Haq H
Arrowsmith S
Moledina J
St George's Hospital , London, UK

159 Enhancing documentation through an electronic clerking proforma for hand injuries in a major trauma centre – a multi-cycle audit

Haq H
Yip S
Moledina J
St George's Hospital, London, UK

161 Morbidity and mortality in surgical simulation: a quality improvement project

Prakash A
Bathla S
St Helens and Knowsley Teaching Hospital NHS Trust , St Helens, UK

162 Using integrated patient leaflets and consent forms to better aid patient understanding of oncoplastic breast surgery: a quality improvement project

Prakash A
Bathla S
St Helens and Knowsley Teaching Hospitals NHS Trust , St Helens, UK

170 Assessment of the management of hand injuries sustained by animal bites at a plastic surgery unit

Bakko F
Song M
Harsten R
Jatan A
Chelsea & Westminster Hospital Plastic Surgery Department, London, UK

172 Adherence to guidelines in surgically managing patients presenting with pancreatitis secondary to gallstones

Bald A
Belhasan A
Gateshead NHS Foundation Trust, Newcastle, UK

175 Could using video otoscopy in audiology departments help cut NHS waiting times and costs?

Hocknell E
Mitchell-Innes A
Ralph E
Musgrove Park Hospital , Taunton, UK

191 A quality improvement project (QIP) assessing suspected scaphoid fracture imaging modality choice against national guidance (NICE/BOA)

Hausien O
Zuberi S
Abdul Z
Mathew P
McArthur G
Chelsea Westminster, London, UK

193 An audit to assess elective general surgery day case rates at a local district general hospital against national guidelines (BADS)

Zuberi S
Saleem RJ
Elkomos B
Zafar N
Northwick Park Hospital , London, UK

197 Assessing adherence to BOAST guidelines in the management of paediatric forearm fractures

Pattnaik S
Khalid M
Sidhu G
Punwar S
Lewisham and Greenwich NHS Trust, London, UK

198 The implementation and departmental benefits of urethral s-curved coaxial dilatation as an adjunct at flexible cystoscopy during the COVID-19 pandemic

Grounds J
Anjana R
Potter H
Karayi M
Sengupta A
McLoughlin J
Gordon E
Dev H
Wilson G
West Suffolk Hospital , Bury St. Edmunds, UK

201 Simulated training session for foundation doctors reduced delays in Mitomycin-C administration

Dereham A 1
Steele J 2
Ralph A 1
1 Royal Lancaster Infirmary, Lancaster, UK
2 Medac Pharma, Lancaster, UK

203 Improving the contactability of the orthopaedic team in a firm based department: a 2-cycle closed loop quality improvement project

Brooker-Thompson C
Adams H
Chelsea and Westminster Hospital , London, UK

206 The implementation of digital operative notes and impact on quality in line with Royal College of Surgeons of England guidance on good surgical practice

Wilson H
Royal Free Hospital , London, UK

207 Surgical mentoring – what can we offer as surgical trainees to medical students

Sivakumar N 1
Wu TW 2
Soupashi M 1
Brace LA 3
Hundle A 4
Jesani H 5
1 George Eliot Hospital , Coventry, UK
2 Leicester University Hospital Trust , Leicester, UK
3 University Hospital Coventry Warwickshire , Coventry, UK
4 University Hospital Leicester , Leicester, UK
5 Universiy Hospital Birmingham , Birmingham, UK

214 The introduction of a pre-incision checklist improves theatre productivity in the management of open lower limb fractures at a major trauma centre: a closed loop audit

Lacey H
Bernard K
King I
University Hospitals Sussex NHS Foundation Trust , Brighton, UK

216 Audit: neurovascular status documentation for paediatric supracondylar fractures

Ahmed T
Shah V
Royal Stoke University Hospital , Stoke on Trent, UK

217 Audit: neurovascular status documentation for paediatric forearm fractures

Ahmed T
Shah V
Royal Stoke University Hospital , Stoke on Trent, UK

218 Audit of variability in booking elective skin procedures

Nordin MNM
Fesatidou V
King I
Queen Victoria Hospital , East Grinstead, UK

219 Documentation and adherence to appropriate margins for BCC and SCC in head and neck sites

Nordin MNM
Ward J
King I
Queen Victoria Hospital , East Grinstead, UK

221 Audit on antibiotic prophylaxis and post-procedure complication rate for patients undergoing transperineal template biopsies of the prostate

Hajuthman W 1 2
Warner R 2
Bodiwala D 2
Shahinur R 3
Abraham M 2
Helliwell H 2
1 Sherwood Forest Hospitals Trust , Mansfield, UK
2 Nottingham University Hospitals NHS Trust , Nottingham, UK

222 A closed loop audit of otitis externa management in primary care: are we over-prescribing oral antibiotics?

Nordin MNM
Dranova S
Goddard N
Robson T
Verkerk M
Grammatopoulou V
Royal Surrey County Hospital , Guildford, UK

225 Delivery of service in urology two weeks’ wait clinic for haematuria

Hossain MA
Cheltenham General Hospital , Cheltenham, UK

226 Diagnosis challenge of necrotising fasciitis: Can we diagnose early? – A quality improvement project at Kettering General Hospital

Swealem A
Basha A
Shyamsundar S
Kettering General Hospital , Kettering, UK

228 An audit on urea and electrolytes checks in patients presenting with acute urinary retention with high residual

Hossain MA
Shaheed Suhrawardy Medical College Hospital , Dhaka, Bangladesh

230 An audit on timely exploration of probable testicular torsion patients

Hossain MA
Shaheed Suhrawardy Medical College Hospital , Dhaka, Bangladesh

233 Postoperative pneumonia following emergency laparotomy

Ho NX
Parmar D
Belhasan A
Queen Elizabeth Hospital , Gateshead Health NHS Foundation Trust, Gateshead, UK

242 Cancellation of elective vascular surgery on a scheduled day in a tertiary hospital

Miah M
Avabde D
Batchelder A
Sidloff D
Nottingham University Hospital , Nottingham, UK

247 Preoperative group and save for cholecystectomies: a necessity or a waste of resources?

Chang LMY
Wilkins A
Hull University Teaching Hospitals NHS Trust , Hull, UK

249 An audit on the length of stay of hip and knee arthroplasty in an elective orthopaedic unit

Patel J 1
Ahmed S 2
1 University of Oxford , Oxford, UK
2 Maidstone and Tunbridge Wells NHS Trust, Kent, UK

251 Emergency laparoscopic cholecystectomy: audit of a local experience

Adenipekun A
Shalaby AI
Wiggins T
Stamatiou D
University Hospitals Birmingham NHS Foundation Trust , Birmingham, UK

252 Accuracy of clinical coding in elective skin cancer procedures – a quality improvement project

Turnbull C 1
Aslam S 2
Maamoun W 2
1 NHS Lothian, Edinburgh, UK
2 University Hospital of North West Midlands , Stoke-on-Trent, UK

257 A clinical audit of antibiotic prescription review in the orthopaedic department at King's College Hospital

Worrall A 1 2
Lister K 1
1 King's College Hospital , London, UK
2 Croydon University Hospital , London, UK

276 Improving the efficiency of ENT emergency clinic in a climate of staffing shortages and junior strikes

Meredith E
Anbu D
Benjamin E
Charing Cross Hospital , London, UK

Subjects: Robotics and Digital Surgery

67 Beyond the scalpel: augmented reality reshaping post-graduate surgical skill training

Aloul Z 1
El-Bahnasawi M 2
Colman S 3
Abdulkader N 4
Luthra P 5
Brown J 5
Rawaf D 6
1 Cardiff University School of Medicine , Cardiff, UK
2 Wythenshawe Hospital , Manchester University Foundation Trust , Manchester, UK
3 Manchester University NHS Foundation Trust , Manchester, UK
4 Southern Hospital , Mid Essex NHS Trust, Essex, UK
5 Edge Hill University , Lancashire, UK
6 Inovus Medical, London, UK

88 Robotic assisted kidney transplant vs conventional open kidney transplant for end stage renal disease. Comparison of perioperative graft and patient outcome: a systematic review

Rajasekar V
Mehta T
Patel B
Barts Cancer Institute , London, UK

262 Robotic-assisted knee arthroplasty: a comprehensive review of efficacy, costs, and future perspectives

Yasen Z
Imperial College London , London, UK

272 Development of a 3-dimensional printing pathway for preoperative planning in complex craniofacial reconstruction

Rossiter M 1 2 3
Jasionowska S 1 2 3
Goble M 3
Ponniah A 2 3
Butler P 2 3
1 University College London , London, UK
2 Royal Free Hospital , London, UK
3 Charles Wolfson Centre , London, UK

Subjects: Systematic Review and Meta-Analyses

13 An overview of laparoscopic surgical training modalities – how does augmented reality simulation compare?

Ludick C 1
Rawaf D 2
Swealem A 3
1 Nottingham Trent University , Nottingham, UK
2 Inovus Medical, Saint Helens, UK
3 Kettering General Hospital , Kettering, UK

22 The efficacy of antiseptic treatment of surgical drains on bacterial colonisation and surgical site infection post breast surgery: a systematic review and meta-analysis

Taha N
Rahman S
Kilshaw A
Leeds General Infirmary, Leeds, UK

30 Breast invasive carcinoma in children and teenagers: a narrative literature review

Hassan N
Alkistawi F
Qavi Q
Abdalla Al-Zawi A Saad
Basildon and Thurrock University Hospital , Essex, UK

44 Investigating the efficacy of Cyberknife to improve survival in advanced pancreatic cancer

Mehta A 1
Awuah WA 2
Kumar H 3
Sivanandan J 4
Adebusoye FT 2
Roy S 5
Shah S 6
Bharadwaj H 7
1 University of Debrecen , Debrecen, Hungary
2 Sumy State University , Sumy, Ukraine
3 Dow University of Health Sciences , Karachi, Pakistan
4 SRM Medical College Hospital and Research Centre , Kattankulathur, India
5 Queen's University Belfast , Belfast, UK
6 Royal College of Surgeons in Ireland , Dublin, Ireland
7 The University of Manchester , Manchester, UK

45 Outcomes for prostate cancer patients undergoing penile prosthesis insertion following pelvic surgery and radiotherapy

Bharadwah HR 1
Javed M 2
Bone M 1
Pang K 3
Alnajjar H 3
1 The University of Manchester , Manchester, UK
2 University of Nottingham , Nottingham, UK
3 Institute of Andrology , University College London Hospitals NHS Foundation Trust , London, UK

46 Cranial meningioma resection by endoscopic surgery: a comprehensive systematic review

Bharadwaj HR 1
Javed M 2
Tan JK 3
Choudhry A 4
Mehta K 5
Awuah WA 6
Wellington J 7
Dalal P 8
1 The University of Manchester , Manchester, UK
2 University of Nottingham , Nottingham, UK
3 University of St. Andrews , St. Andrews, UK
4 John Radcliffe Hospital , Oxford University Hospitals Trust , Oxford, UK
5 GMERS Medical College , Vadodara, India
6 Sumy State University , Sumy, Ukraine
7 Bradford Teaching Hospitals NHS Foundation Trust , Bradford, UK
8 University of Central Lancashire , Preston, UK

52 The potential of mental rehearsal in surgical education: a comprehensive review of the evidence

Walshaw J 1
Huo B 2
Banks P 3
McClean A 3
Mushtaq F 4
Jayne D 1
Miskovic D 5
Yiasemidou M 6
1 Leeds Institute of Medical Research , St James’s University Hospital , University of Leeds , Leeds, UK
2 Dalhousie University , Halifax, Canada
3 Bradford Teaching Hospitals NHS Foundation Trust , Bradford, UK
4 School of Psychology , University of Leeds , Leeds, UK
5 St Mark's, London North West University Healthcare , London, UK
6 Oxford University Hospitals NHS Foundation Trust , Oxford, UK

61 Bilateral breast calciphylaxis: a case report and literature review

Hassan N
Chicken W
Rashid N
Idaewor P
Hughes T
Alkistawi F
Qavi Q
Al-Zawi AS Abdalla
Basildon and Thurrock University Hospital , Essex, UK

65 Optimal venous anastomoses in traumatic lower limb free flap reconstruction: evaluating single vs dual approaches

Stark D 1
Yim G 2
1 North Bristol NHS Trust, Bristol, UK
2 Swansea Bay University Health Board , Swansea, UK

70 Current state of minimally invasive general surgical practice in Africa: a systematic review and meta-analysis of the laparoscopic procedures performed and outcomes

Falola A 1 2
Dada O 1 3
Akande D 1 2
Adenikinju J 1 4
Fadairo R 1 2
Ogbodu E 1 5
Effiong-John B 1 2
Adelotan A 1 6
Ndong A 1 7
1 General Surgery Community, Surgery Interest Group of Africa, Lagos, Nigeria
2 University of Ibadan College of Medicine , Ibadan, Nigeria
3 University Hospitals Birmingham NHS Foundation Trust , Birmingham, UK
4 London North West University Healthcare NHS Trust , London, UK
5 Asaba Specialist Hospital , Asaba, Nigeria
6 University of Port Harcourt , Port Harcourt, Nigeria
7 Gaston Berger University , Saint-Louis, Senegal

73 Real-time assessment of surgical team performance in operating rooms: a systematic review on state-of-the-art

Gogoi P
Mylonas G
Kinross J
Imperial College London , London, UK

75 Beyond the scalpel: lymphedema considerations in breast cancer treatment

Stark D
North Bristol NHS Trust, Bristol, UK

85 Air vs fluorinated gas tamponades in pars plana vitrectomy for rhegmatogenous retinal detachment: systematic review and meta-analysis

Alsaif A 1
Karam M 2
Alabdulialil T 3
Kahlar N 4
Shareef E 5
Alzaid M 6
Alotaibi A 5
1 Kingston Hospital Foundation Trust , London, UK
2 McGill University , Montreal, Canada
3 Ibn Sina Hospital , Ministry of Health, Kuwait; Kuwait University , Jabriya, Kuwait
4 Sandwell and West Birmingham Hospitals NHS Trust , Birmingham, UK
5 Ibn Sina Hospital , Ministry of Health, Kuwait
6 School of Medical Sciences , University of Manchester , Manchester, UK

113 Evaluating the surgical outcomes of robot-assisted simultaneous resection of colorectal carcinoma with synchronous liver metastasis vs laparoscopic approach: a systematic review

Mehta T
Rajasekar V
Patel B
Queen Mary University , London, UK

121 Prehospital tranexamic acid in trauma patients: a systematic review and meta analysis of randomised controlled trials

Acharya P
East and North Hertfordshire NHS Trust, Stevenage, UK

130 Is MIDCAB the future of beating heart cardiac surgical interventions for adults with coronary heart disease? A systematic review

Singh S 1 2 3
Patel B 2
1 Barts and the London School of Medicine & Dentistry , London, UK
2 Barts Cancer Institute , London, UK
3 Queen Mary University of London , London, UK

150 Do the current predeployment training programmes of military surgeons enable them to perform effectively on placement to active warzones?

Howard J
University of Liverpool , Liverpool, UK

153 Transforming surgical practice: innovations and trends in the UK - systemic analysis

Siddique MH 1
Bashir M 2
1 QEHB, Birmingham, UK
2 Heartlands Hospital , Birmingham, UK

157 Systematic review of localisation techniques for non-palpable breast tumours

Rezacova M
Winchester Hospital , Winchester, UK

166 Local vs general anaesthesia for transcatheter aortic valve implantation (TAVI): a systematic review, meta-analysis and trial sequential analysis of randomised and propensity-score matched studies

Jaffar Karballai M 1
Al-Tawil M 2
Roy S 3
Kayali F 4
Vankad M 5
Harky A 6 7
Zeinah M 6 8
1 St George's University of London , London, UK
2 Al-Quds University , Jerusalem, Palestine
3 Queen's University Belfast , Belfast, UK
4 University Hospitals Sussex , Sussex, UK
5 University Hospitals Birmingham , Birmingham, UK
6 Liverpool Heart and Chest Hospital , Liverpool, UK
7 University of Liverpool , Liverpool, UK
8 Ain Shams University , Cairo, Egypt

173 Connectome modifications and transcranial magnetic stimulations as neurorehabilitation for stroke patients: a scoping review protocol

Suvarna R 1
Murthy S 2
Datta-Banik R 3
Radhakrishna G 4
Wellington J 5
1 School of Medicine , University of Leeds , Leeds, UK
2 Università Degli Studi di Bari Aldo Moro, Bari, Italy
3 Universidad Marista de Mérida , Mérida, Mexico
4 Osh State University , International Medical Faculty, Osh, Kyrgyzstan
5 Bradford Teaching Hospitals NHS Foundation Trust , Bradford, UK

174 Aortic root replacement vs patch repair for aortic valve endocarditis with root abscesses: a systematic review and meta-analysis of short- and long-term outcomes

Al-Zubaidi F 1
Jaffar-Karballai M 2
Massias SA 3
Kuku D 4
Vijayarasa V 5
Al-Tawil M 6
Harky A 7
1 Royal Papworth Hospital , Cambridge, UK
2 St George’s University of London , London, UK
3 Watford General Hospital , Watford, UK
4 Chelsea and Westminster Hospital , London, UK
5 University of Niš , Niš, Serbia
6 Al-Quds University , Jerusalem, Palestine
7 Liverpool Heart and Chest Hospital , Liverpool, UK

176 A scoping review of clinical presentations and outcomes in patients with concomitant COVID-19 infection and acute mesenteric ischaemia

Cai W 1
Zhao Y 2
Mallappa S 3
1 Colchester General Hospital , Colchester, UK
2 Imperial College London , London, UK
3 West Hertfordshire Teaching Hospitals NHS Trust , West Hertfordshire, UK

182 Laparoscopic ultrasonography during laparoscopic cholecystectomy: systematic review

Awan B 1
Elsaigh M 1
Elkomos BE 1
Sohail A 1
Asqalan A 2
Baqar SOM 3
Elgendy NA 4
Saleh O 5
Szul JM 1
Juan AS 1
Marzouk MM 1 6
Alasmar M 7
1 Northwick Park Hospital , London Northwest Hospital NHS Trust , London, UK
2 Norfolk and Norwich University Hospital , Norwich, UK
3 Derriford Hospital , University Hospital Plymouth , Plymouth, UK
4 Park Hospital , Surrey, UK
5 Brigham and Women’s Hospital , Harvard Medical School , Boston, USA
6 Ain Shams University Hospital , Cairo, Egypt
7 Salford Royal Hospital , University of Manchester , Manchester, UK

183 The use of lidocaine infusion in laparoscopic cholecystectomy: an updated systematic review and meta-analysis

Awan B 1
Elsaigh M 1
Elkomos BE 1
Sohail A 1
Asqalan A 2
Baqar SOM 3
Elgendy NA 4
Saleh O 5
Szul JM 1
Juan AS 1
Alasmar M 6
Marzouk MM 1 7
1 Northwick Park Hospital , London Northwest Hospital NHS Trust , London, UK
2 Norfolk and Norwich University Hospital , Norwich, UK
3 Derriford Hospital , University Hospital Plymouth , Plymouth, UK
4 Frimley Park Hospital , Surrey , UK
5 Brigham and Women’s Hospital , Harvard Medical School , Boston, USA
6 Salford Royal Hospital , University of Manchester , Manchester, UK
7 Ain Shams University Hospital , Cairo, Egypt

190 Is it the time for the sun to set on the unoperated obstructed cancer colon? Updated systematic review and meta-analysis for the management of obstructed cancer colon

Elkomos BE 1 2
Alkomos PE 3
Alkomos MF 4
Elsaigh M 1
Awan B 1
Sohail A 1
Asqalan A 5
Nuno A 1
Zuberi S 1
Rai J 1
Kalakouti E 1
Junaid R 1
Ebeidallah G 6
1 Northwick Park Hospital , London Northwest Hospital NHS Trust , London, UK
2 Ain Shams University , Cairo, Egypt
3 Ain Shams University Hospital , Cairo, Egypt
4 St. Joseph’s University , Paterson, New Jersey, USA
5 Norfolk and Norwich University Hospital , Norwich, UK
6 Royal Derby Hospital , University Hospitals of Derby and Burton NHS Foundation Trust , Derby, UK

229 Comparative analysis of acellular dermal matrices in implant-based breast reconstruction: a systematic review and network meta-analysis

Glynou SP 1
Sousi S 2
Cook H 3
Zargaran D 2 3
Mosahebi A 3 4
1 Queen Mary University of London , London, UK
2 University College London , London, UK
3 Royal Free London NHS Foundation Trust, London, UK
4 University College London , London, UK

260 A scoping review protocol of the current state of video in the use of training of cardiac surgeons in LMICS protocol

Garg S
Maharaj Mojarad S
University of Nottingham , Nottingham, UK

263 Modern surgery and endoscopic procedures for gastro-oesophageal reflux in lung transplant recipients: a systematic review and meta-analysis

Ali I
Namgoong C
Dad B
Robb H
Fehervari M
Imperial College London , London, UK

269 A systematic review of long-term outcomes following Parsonage Turner Syndrome and an evidence-based algorithm to optimise patient management

Al Hinai R 1
Wilkinson J 1
Abdul Jalil L 2
Dolan R 2
1 St Vincent’s University Hospital , Dublin, Ireland
2 Beaumont Hospital , Dublin, Ireland

